# The Paradigms They Are a-Changin’: past, present and future of PVC bacteria research

**DOI:** 10.1007/s10482-017-0962-z

**Published:** 2017-10-20

**Authors:** Elena Rivas-Marín, Damien P. Devos

**Affiliations:** Centro Andaluz de Biología del Desarrollo (CABD)-CSIC, University Pablo de Olavide, Carretera Utrera, km 1, 41013 Seville, Spain

**Keywords:** *Chlamydiae*, Evolutionary cell biology, *Kirimatiellaeota*, *Lentisphaerae*, *Candidatus* Omnitrophica, *Planctomycetes*, *Verrucomicrobia*

## Abstract

These are exciting times for PVC researchers! The PVC superphylum is composed of the bacterial phyla *Planctomycetes*, *Verrucomicrobia, Chlamydiae* (those three founders giving it its name), *Lentisphaerae* and *Kirimatiellaeota* as well as some uncultured candidate phyla, such as the *Candidatus* Omnitrophica (previously known as OP3). Despite early debates, most of the disagreements that surround this group of bacteria have been recently resolved. In this article, we review the history of the study of PVC bacteria, with a particular focus on the misinterpretations that emerged early in the field and their resolution. We begin with a historical perspective that describes the relevant facts of PVC research from the early times when they were not yet termed PVC. Those were controversial times and we refer to them as the “discovery age” of the field. We continue by describing new discoveries due to novel techniques and data that combined with the reinterpretations of old ones have contributed to solve most of the discordances and we refer to these times as the “illumination age” of PVC research. We follow by arguing that we are just entering the “golden age” of PVC research and that the future of this growing community is looking bright. We finish by suggesting a few of the directions that PVC researches might take in the future.

## The early days, before the superphylum: the phyla

**Fig. 1:**
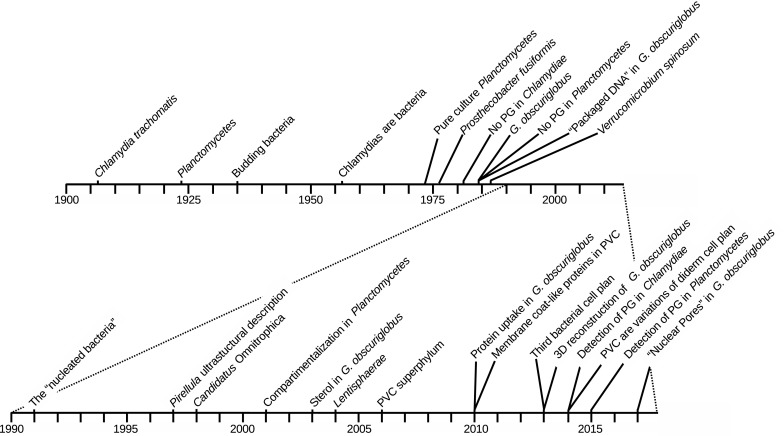
Timeline of the major events in PVC research. A zoom in the decades between 1990 and present is shown

It is only relatively recently that the status of superphylum has been proposed and even more recently that it has been accepted by the community. For this reason, we describe below the early times of each phyla separately (Fig. 1).

### *Chlamydiae*

The very first member of the group to be reported was the one currently known as *Chlamydia trachomatis*. Staining of conjunctival epithelial cells of trachoma patients had shown dark-stained inclusions with dense particles that were originally considered as neither protozoa nor bacteria, but referred to as “*Chlamydozoa*” (from the Greek word χλαμεσ, meaning mantle or cloak) (Von Prowazek and Halberstadter 1907). They were then regarded as viruses, and it was only in 1957 that they were recognized as bacteria and isolated in pure cultures (Tang et al. 1957). Thus, from their very first description, these bacteria were already confusing scientists. Later, these unique microorganisms were found to be amongst the most important bacterial pathogens of humankind (Elwell et al. 2016). Initially containing only pathogenic species, environmental or amoeba symbiotic organisms have been recently described [reviewed in Horn 2008]. Because those are non-pathogenic but share fundamental cell biology processes with the pathogenic ones, they form excellent model systems to decipher the molecular bases of this historical plague to society (Seth-Smith et al. 2017; König et al. 2017).

### *Planctomycetes*

In 1924, a free-living rosette-forming organism was described in freshwater ponds in Budapest, Hungary (Gimesi 1924). The cells were bigger than most bacteria known by then and even by now. Their appearance was also uncommon for bacteria, having a different morphology composed by several spherical cells stalked with a holdfast at its tip that attaches them to form a microcolonial rosette. As a result, they were confused with fungi and named *Planctomycetes*, which means “floating fungi”. Thus, here again, the confusion started early. The first species described was *Planctomyces bekefii*. Arthur T. Henrici was the first bacteriologist to describe members of the *Planctomycetes* as bacteria (Henrici and Johnson 1935). Despite this early start, the first isolate of the phylum, *Pirellula staleyi*, was not described until the 1970s (Staley 1973; Schmidt 1978). The planctomycete *Gemmata obscuriglobus* was isolated from the freshwater Maroon Dam (Queensland, Australia) in 1984, and described as containing “packaged nuclear material” (Franzmann and Skerman 1984). It has since then become one of the most studied planctomycetal strains because of its unusual cell biology, including a developed endomembrane system, a major feature of this phylum (Devos 2013). *Planctomycetes* also contains the anammox bacteria, the only ones able to achieve the unique reaction of anaerobic ammonium oxidation (Strous et al. 1999). Since then, the number of described strains has risen steadily to reach more than 36 (Jogler C., personal communication). Condensed DNA appears to be a common factor of most planctomycetes, a feature shared with other bacteria, such as *Deinococcus radiodurans* (Cox and Battista 2005). Members of this phylum are ubiquitous, mostly found in soil, sea or fresh water, and mainly free-living (Hackl et al. 2004; Polymenakou et al. 2005; Lindström et al. 2005; Sangwan et al. 2005; He et al. 2006).

### *Verrucomicrobia*

*Prosthecobacter* species were originally described as fusiform caulobacter, again deceving us at first (Staley et al. 1976). *Verrucomicrobium spinosum* was the first member of the *Verrucomicrobiales* to be reported in 1987 (Schlesner 1987). Other species were described shortly after but the phylum was, however, not proposed until 1996 (Hedlund et al. 1996). This group contains members of the microbial communities from soil, fresh and marine water, as well as symbionts of nematodes or of the human guts.

### *Lentisphaerae*

The *Lentisphaerae* phylum was proposed in 2004 after the isolation of two marine strains (Cho et al. 2004). Member of this phylum are widely distributed, being present in seawater and sediments (Giovannoni and Stingl 2005; Polymenakou et al. 2009), anaerobic sludge (Schlüter et al. 2008; Rivière et al. 2009) and landfill leachate (Limam et al. 2010). This phylum also comprises the former candidate phylum VadinBE97 and several organisms that live in association with eukaryotes (Myer et al. 2015; Passarini et al. 2015; Niu et al. 2015) as well as *Victivallis vadensis*, a strictly anaerobic cellobiose-degrading isolate from human faeces (Zoetendal et al. 2003).

### The other phyla

The *Candidatus* Omnitrophica was isolated in 1998 from Obsidian Pool in the Yellowstone National Park (Hugenholtz et al. 1998). A magnetotactic representative from this group, *Candidatus* Omnitrophus magneticus SKK-01, was reported as the first magnetotactic member of the PVC superphylum (Kolinko et al. 2012). Recently, a novel phylum, *Kirimatiellaeota*, has been proposed as representing a novel phylum of the PVC group (Spring et al. 2016). Members of this new phylum occupy predominantly niches characterized by anoxic conditions, like the intestine of animals, wastewater or hypersaline sediments, whereas few representatives have been detected in soil or the water column of marine and freshwater habitats. These phyla are particularly poorly described.

## The controversies and their resolution

Early on, various conflicting interpretations have been presented in the field. The times of resolution, the “illumination age” of PVC research, approximately covers the year 2005–2015. Below we describe those controversies and their resolutions.

### Superphylum

A major issue for the field was the status of the superphylum, as its absence impeded the recognition of shared ancestry and thus of shared cell biology between these bacteria. Despite very different lifestyles and phenotypes, that do not suggest a common origin, early 16S rDNA and ribosomal RNA based phylogenetic methods suggested that the *Planctomycetes* and *Chlamydiae* were the closest relatives of the *Verrucomicrobia*; nevertheless, bootstrap support for such associations were weak (Albrecht et al. 1987; Ward-Rainey et al. 1995; Hedlund et al. 1996). This was supported by analysis of the 16S rRNA sequence of the anammox (Strous et al. 1999) and by phylogenies, based on rRNA, which placed *Verrucomicrobia* and *Lentisphaera* in proximity to *Planctomycetes* and *Chlamydiae* (Cho et al. 2004). The superphylum was however not recognized by all (Ward et al. 2000; Jenkins and Fuerst 2001; Ciccarelli et al. 2006). Evidence that those phyla might be more related than previously suspected slowly accumulated and formal proposal of the superphylum was presented in 2006 (Wagner and Horn 2006). Therefore, we take the year 2006 as the one of the birth of the superphylum, the beginning of the “illumination age” of PVC research. Later, this proposal received additional support from genomic and phylogenetic analyses of conserved proteins (Griffiths and Gupta 2007; Hou et al. 2008; Pilhofer et al. 2008; Gupta et al. 2012; Kamneva et al. 2012).

Although, the status of the superphylum is now mostly accepted, its extent is still debated. For instance, *Poribacteria*, detected in 2004 in marine demosponges (Fieseler et al. 2004), was proposed to be part of the PVC superphylum in 2006 (Wagner and Horn 2006) but recent studies based on single genomes instead proposes that they are a sister phylum to the PVCs (Gupta et al. 2012; Kamke et al. 2014). Similarly, isolate WWE2 from a municipal anaerobic sludge digester was initially proposed to represent a novel candidate phylum (Chouari et al. 2005), however, more recent analysis has placed it as a subdivision of the *Lentisphaerae* (Limam et al. 2010). The PVC superphylum has also been proposed to be part of a “megaphyla” containing the *Spirochaetes*, the *Chlorobi*-*Bacteroidetes* group, *Gemmatimonadetes* and *Fibrobacteres* (Yutin et al. 2012).

**Fig. 2:**
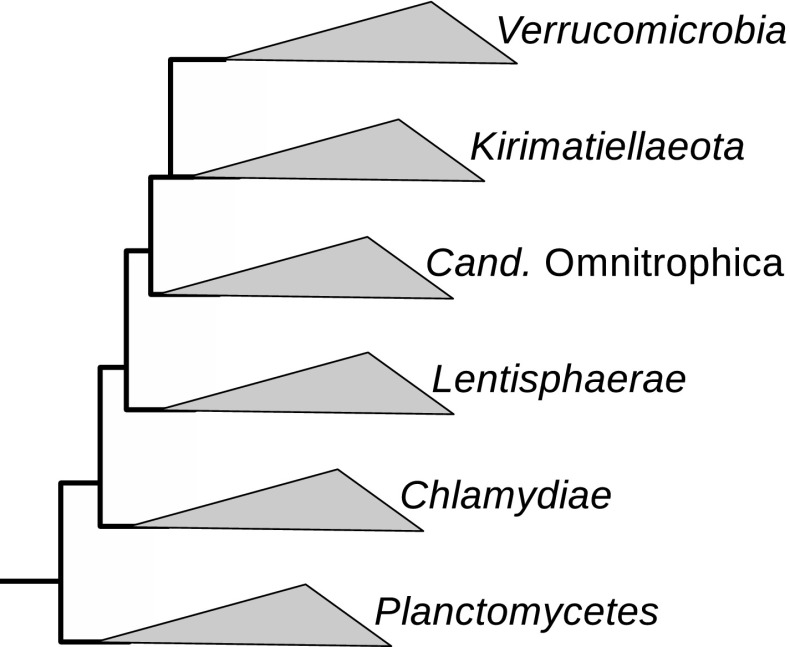
Phylogenetic representation of the PVC superphylum based on 16S rRNA gene sequences and rooted on the *Escherichia coli* K12 sequence [adapted from (Spring et al. 2016)]. This tree is supported by RpoB protein based phylogenies and by the phylogenetic composition of the genomes of several representative species. Although still putative, this is the current most complete and comprehensive tree of the PVC superphylum

Relationships between the phyla have been differently described, but the phylogenies are converging with new data (Fig. 2). More analyses on the extent of the PVC superphylum and phylogenetic relationships of its members are needed.

### Third bacterial cell plan

Another confusing factor of these bacteria was the proposed alternative cell plan of some of them. This interpretation was based on the observed development of their intracellular membranes, particularly expanded in some members of the *Planctomycetes*. In 1991, a double membrane that seemed to surround the genetic material was described in electron micrographs of the planctomycete *G. obscuriglobus*. The extrapolation of these two-dimensional images into a compartment defined by membranes in three-dimensions was claimed to fill the gap between prokaryotes and eukaryotes. It was concluded that “The occurrence of a membrane-bounded nucleoid in a eubacterial prokaryote is a significant exception to the evidence supporting the prokaryote/eukaryote dichotomous classification of cell structure” (Fuerst and Webb 1991).

In 2001, this view was extended to other planctomycetes and it was proposed that their membrane organization defined a new cell plan previously unseen in bacteria (Lindsay et al. 2001). The title of this publication says it all: “Cell compartmentalisation in planctomycetes: novel types of structural organization for the bacterial cell”. The cell organization of various planctomycetal species, including *G. obscuriglobus*, was investigated based on ultrastructure derived by cryo-electron microscopy. This cellular organization was proposed to be different from the classical diderm (Gram-negative) or monoderm (Gram-positive) bacteria and to define a new cell plan. This particular cell plan was also claimed to be present, although with differences, in members of the *Verrucomicrobia* (Lee et al. 2009).

In planctomycetal and verrucomicrobial cells, the outermost membrane was identified with the cytoplasmic membrane (CM). All internal membranes were identified as intracellular membranes (ICM) and invaginations thereof. Because of the new identities of the membranes, the volumes they defined inside the cell also had to be reclassified. The most internal volume, containing ribosomes was termed the riboplasm, while the space contained between the ICM and the CM, devoid of ribosomes, was called the paryphoplasm. This third bacterial cell plan hypothesis dominated the field uncontested for some years.

Later, it was realized that most planctomycetal and verrucomicrobial genomes encode proteins that are typical of the outer membrane of diderm bacteria (Strous et al. 2006; Speth et al. 2012). Around the same time, it was argued, based on the first complete three-dimensional reconstructions of a bacterium, that there is no membrane delimited intracellular compartments in *G. obscuriglobus* cells, other than the cytoplasm and periplasm as in classical diderm bacteria (Santarella-Mellwig et al. 2013). The endomembrane system is extremely developed, resulting in a three-fold increase of the internal membrane surface as compared to classical bacteria. But there are no closed compartments inside the cytoplasm, the membrane invaginations never completely surround a defined cellular volume. In particular, there is no double membrane completely surrounding the genetic material, and thus no “nucleated bacteria”. The only exceptions to this claim seems to be present in the anammox bacteria, in which a membrane defined compartment, similar to a eukaryotic vacuole, could be isolated from the cells. This particular compartment is related to the peculiar anammox reaction (Neumann et al. 2014).

Based on the gathering of previous evidence and on the reconsideration of old data, it was then proposed that the various forms of endomembrane systems found in PVC members are variation of, but no exception to, the diderm cell plan (Devos 2014a). Likely causes of previous misinterpretations have been presented (Devos 2014b). Lipopolysaccharide, a signature of the classical diderm outer membrane was also reported in *G. obscuriglobus*, further supporting the diderm cell plan (Mahat et al. 2015). This cell plan is now accepted by the vast majority of members of the community. Despite this, alternative interpretations are still presented (Sagulenko et al. 2017; Feijoo-Siota et al. 2017).

### No PG for PVCs, at least for some of them

Another misleading claim of *Chlamydiae* and *Planctomycetes* was the belief that they lacked peptidoglycan (PG). Despite the fact that PG is an almost universal feature of the bacterial cell wall, it had not been detected biochemically in both phyla in early analyses (Garrett et al. 1974; Caldwell et al. 1981; Barbour et al. 1982; König et al. 1984; Liesack et al. 1986; Fox et al. 1990; Ghuysen and Goffin 1999). The cell wall of various planctomycetes was instead found to be mostly composed of proteins (Stackebrandt et al. 1986). Visualization of thin sections with the technologies available at the time, also supported this absence, as no intermediary layer was observed between the two membranes (Tamura et al. 1971; Huang et al. 2010). Thus, members of the phyla *Chlamydiae* and *Planctomycetes* were both claimed to be deprived of PG, which was an additional and important argument of the third cell plan interpretation. Surprisingly, and contrarily to planctomycetes, chlamydias are susceptible to β-lactam antibiotics (Matsumoto and Manire 1970; Cayrou et al. 2010). What’s more, some of the PG synthesis genes were detected and even shown to be functional in some members of the *Chlamydiae* (McCoy et al. 2003; Hesse et al. 2003; McCoy and Maurelli 2005; Patin et al. 2009, 2012). This conundrum was named “the chlamydial anomaly” (Moulder 1993).

Access to complete genomes did not immediately resolve this controversy. Indeed, in most model bacteria, the *dcw* (division and cell wall) genes are well conserved and clustered in a small locus of the genome containing most of the genes involved in cell division or PG synthesis. This includes the *fts* (for filamentous temperature sensitive) genes involved in cell division and the PG precursors synthesis genes. In agreement with the absence of PG detection in *Chlamydiae* and *Planctomycetes*, the first analyses of complete genomes failed to detect some of the key genes in the *dcw* cluster of some PVC members, in particular in most planctomycetal and chlamydial genomes (Pilhofer et al. 2008; Jogler et al. 2012). This reinforced the belief that *Planctomycetes* and *Chlamydiae* had no peptidoglycan because they had lost some of the key *dcw* genes.

This abnormality has been more recently re-addressed using a more sensitive method, revealing that the planctomycetal and chlamydial species investigated carry most of the genes required for PG synthesis. In agreement with the diderm derived cell plan of the PVC bacteria, PG was detected in some members of the *Chlamydiae* (Pilhofer et al. 2013; Liechti et al. 2014) and the *Plantomycetes* (Jeske et al. 2015; van Teeseling et al. 2015). PG was detected in five planctomycetal strains: *G. obscuriglobus*, *Planctopirus limnophila*, *Rhodopirellula baltica*, strain L21-Rpul-D3 and *Cand.* Kuenenia stuttgartiensis. PG was also observed in *Protochlamydia amoebophila*, a symbiotic member of the *Chlamydiae*. Moreover, novel techniques allowed the determination that PG was indeed synthesized in the pathogenic *C. trachomatis*, but only during division and exclusively at the septum (Liechti et al. 2016). Thus, PG synthesis is tightly regulated in *Chlamydiae*, both in time and spatially, explaining why it had not been detected previously. The presence of PG in at least one member of all PVC phyla definitively concludes the third cell plan controversy, stably anchoring these bacteria in the diderm division of bacteria.

## The controversy till 2015

Up to roughly 2015, the PVC superphylum appeared to bring together very different bacteria. These bacteria had very different phenotypes, from intracellular pathogens to free-living, anaerobic to aerobic, heterotrophs to autotrophs. They also appeared to have seemingly very different cell plan, with some clear diderm (*Verrucomicrobia* and *Lentisphaera*) and some proposed to present a third cell type (*Planctomycetes*). PG has been observed in some bacteria while others were thought to lack it. Additional confusing issues include their division mode, the fact that some bacteria divide by what appears as the “classical” binary fission using a FtsZ centered mechanism, while others have lost many division proteins and some even divide by budding (Rivas-Marín et al. 2016a). In addition, they also contain the anammox planctomycetes with the very unique process of anaerobic ammonium oxidation. Thus, all considered, the PVC superphylum looked like a melting pot of unusual bacteria with little to nothing in common.

It is now well established, that PVC bacteria are a peculiar group of bacteria that are firmly anchored in the diderm bacterial division. Some have expanded their membrane systems or have modified the quantity and time of synthesis of their PG, but all are variations of, and no exception to, the diderm cell plan.

**Fig. 3:**
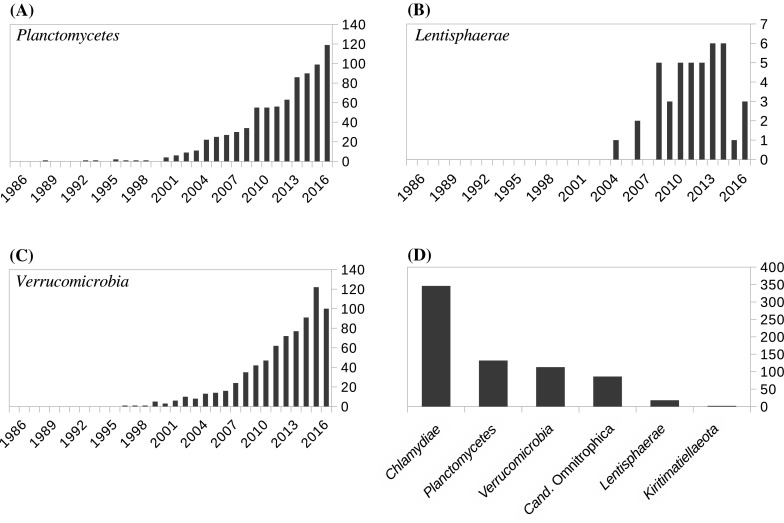
Publication and genome numbers for the PVC phyla **a** *Planctomycetes*, **b** *Lentisphaera* and **c** *Verrucomicrobia* (from Alexandru Dan Corlan. Medline trend: automated yearly statistics of PubMed results for any query, 2004. Web resource at URL: http://dan.corlan.net/medline-trend.html). *Chlamydiae* is not represented due to the huge number of hits related to their pathogenicity. **d** The number of genomes projects as currently recensed at NCBI genomes (https://www.ncbi.nlm.nih.gov/genome/browse/) is displayed

It was around the time of the resolution of these controversies that the PVC community began to gain momentum. The first journal research topic entirely dedicated to bacteria from this superphylum was put together in 2012–13 (Ward NL; http://journal.frontiersin.org/researchtopic/491/recent-advances-in-the-biology-of-planctomycetes-and-verrucomicrobia). The title of this research topic “Recent advances in the biology of planctomycetes and verrucomicrobia” illustrates that the status of superphylum had not entirely pervaded the field just yet. At the same time, 2013, the first conference solely dedicated to the PVC superphylum took place. A journal special issue reporting papers from this first workshop was put together (Devos et al. 2013). Since then, the PVC community has met every other year and another journal research topic, this time on the PVC superphylum, has been published (van Niftrik and Devos 2017). The present publication is part of another special issue dedicated to this superphylum (Devos and Lage 2018). Highlighting this increasing interest in the PVC bacteria, the number of publications dedicated to these bacteria has risen in the last decade (Fig. 3). There are now 735 ongoing genome projects at various stages of completion for the PVC subgroup (as of 08/08/2017 at https://www.ncbi.nlm.nih.gov/genome/browse/). Of these, 346 are from the *Chlamydiae*, 132 from the *Planctomycetes*, 113 from the *Verrucomicrobia*, 18 from the *Lentisphaerae,* 86 from the *Candidatus* Omnitrophica, two from the *Kiritimatiellaeota*, and a few from “unclassified” members of the PVC group.

## Where do we stand now?

We believe that now that most of the controversies have been solved, we are facing the “golden age” of PVC research. The prospect of deciphering of the molecular and cellular biology of these bacteria is more exciting than ever. Considering their features in an evolutionary cell biology framework would be important to understand the forces that have led the evolution of these divergent bacteria (van Niftrik and Devos 2017). Moreover, genetic tools have recently been reported in various organisms providing an ample spectrum of characteristics to interrogate across the phylogenetic tree (Domman et al. 2011; Rivas-Marín et al. 2016b). There is however a need to amplify both the type of tools and the number of species where they can be applied. On the other hand, PVC bioinformatics is still in its infancy and in silico analyses of their features are urgently needed.

### Other PVC peculiarities

Members of the PVC superphylum also show a variety of unusual characteristics. Amongst these, the following are likely to be the subject of future research.

#### Diversity

PVC bacteria appear to be ubiquitous, being present in aquatic (fresh and marine water) and terrestrial habitats as pathogenic, environmental or symbiotic organisms. They have been detected in many different environments such as acidic *Sphagnum*-dominated wetlands (Ivanova and Dedysh 2012), crustacean shell (Kohn et al. 2016), in deep-sea hydrothermal vents (Ding et al. 2017), in the cotton rhizosphere (Qiao et al. 2017), in thermoacidic environments (Dunfield et al. 2007; Pol et al. 2007; Islam et al. 2008), in arsenic contaminated groundwater (Das et al. 2017), in sub-arctic ecosystems (Ivanova et al. 2016), in wastewater treatments plants (Chouari et al. 2003), associated with macroalgae (Bengtsson and Øvreås 2010; Bondoso et al. 2017), and in the human gut (Derrien et al. 2004), amongst others. Planctomycetal and verrucomicrobial sequences are frequently detected in 16S rRNA clone libraries from environmental microbial communities. However, their abundance might have been underestimated primarily because of the universal primers usually used to accomplish this task (Hugenholtz et al. 1998; Bergmann et al. 2011; Cai et al. 2013). The use of more specific primers or of direct sequencing has already contributed to the resolution of this issue (Wang et al. 2015). Another reason is the particular conditions required to isolate them; more specific protocols are already addressing this point (Kohn et al. 2016) but clearly more work in this area is needed and ongoing.

#### Membrane system

One of the peculiar features of PVC bacteria is their endomembrane system, particularly developed in some species. What is the dynamic and regulation of the diverse forms of endomembrane system observed in the same species? What are the causes and consequences of the differences between species?

In the year 2010, it was revealed that some planctomycetal and verrucomicrobial genomes contained genes coding for proteins that are structurally related with membrane coat proteins that are otherwise only observed in the eukaryotic endomembrane system and involved in its formation and maintenance (such as clathrin, COPII’s Sec31 or some nucleoporins) (Santarella-Mellwig et al. 2010; Acehan et al. 2014). In addition, it was shown that one of those bacterial proteins in *G. obscuriglobus* is in close contact with the intracellular membrane, like its eukaryotic counterparts. Deciphering the role of the membrane coat-like protein, their partners and mode of action, in this unique prokaryotic endomembrane system, is a fascinating area of study for the years to come.

At the same time, the ability to internalize whole proteins before their intracellular degradation was reported in the same species. This process is energy-dependent, receptor mediated and one of the proteins bearing structural similarity to the eukaryotic membrane coats was found to be involved. These observations make it resemble eukaryotic endocytosis (Lonhienne et al. 2010). This observation has recently been repeated in *G. obscuriglobus* and extended to another planctomycetes, *P. limnophila* (Boedeker et al. 2017). What is the extent of the similarities and differences with the eukaryotic process is still unknown. Understanding the molecular basis of this process will reveal fascinating knowledge with important evolutionary implications.

Nuclear pore-like structures have recently been described in *G. obscuriglobus* (Sagulenko et al. 2017). What is their role? Are they linked to the phenomenon of protein internalization? Are there related structures in other Planctomycetes or PVC members? More work is needed on these interesting structures.

Sterols are lipids mostly found in eukaryotic membranes and only in a few bacteria. They are essential in eukaryotes and are involved in many cellular processes like signaling as well as in the organisation of the membranes. Most planctomycetes synthesize hopanoids, the bacterial equivalent to sterol. *G. obscuriglobus* is the only planctomycete that produces sterol and hopanoids have not been identified in its membranes so far. It has been proposed that *G. obscuriglobus* could retain the most ancient remnants of the sterol biosynthetic pathway. The evolutionary origin of sterol in this organism, due to lateral gene transfer (LGT) or other, is still debated (Pearson et al. 2003; Chen et al. 2007; Desmond and Gribaldo 2009; Gold et al. 2017). Again, fascinating biology is bound to emerge here.

*Division mode.* The vast majority of bacteria divide by binary fission, a mechanism in which the central player is the FtsZ protein that interacts with the PG biosynthesis machinery. FtsZ and PG are almost ubiquitous in bacteria. PG synthesis is extremely regulated during division in pathogenic chlamydias as it is restricted to a thin ring of PG at the septum present only at the time of division (Liechti et al. 2016). Most PVC bacteria are deprived of the FtsZ protein with the exception of the *Verrucomicrobia* and *Cand.* Omnitrophica. How bacteria divide without FtsZ is still one a significant unknown of microbiology and evolutionary biology. In addition, PVC members present a huge diversity of division modes (Rivas-Marín et al. 2016a). Some species, such as the members of the order *Planctomycetales*, divide by budding, whereas most others divide by binary fission. *Chlamydiae* has historically been described as dividing by binary fission, however, recent analyses have revealed an asymmetric division in *C. trachomatis* (Abdelrahman et al. 2016). It is important to keep exploring the diversity of PVC members in order to expand our knowledge division modes and identify intermediary species and phenotypes (Kohn et al. 2016).

#### Tubulin

Another peculiarity of these bacteria is the presence of tubulin, a eukaryotic landmark protein. The protein constituents of the microtubule cytoskeleton are found in all eukaryotes but have rarely been found in bacteria or archaea. Tubulin had been found previously in other prokaryotes, but those cases are the result of LGT; the phylogenies clearly and stably located the tubulins inside the eukaryotic clades and they have similar biochemical characteristics. One of the few bacterial exceptions is the bacterial verrucomicrobial genus *Prosthecobacter* (Petroni et al. 2000; Jenkins et al. 2002). The presence of these proteins has initially been deemed to be the result of LGT from a eukaryote (Jenkins et al. 2002; Sontag et al. 2005; Schlieper et al. 2005; Pilhofer et al. 2007). However, other recent phylogenies have argued against this conclusion (Pilhofer et al. 2011). In addition, in some phylogenies, the verrucomicrobial tubulin branches basal to the eukaryotic ones and has different biochemical characteristics. In addition to some divergent sequence signal, the verrucomicrobial tubulin polymerizes as a 5-mere protofilament when the eukaryotic ones polymerize as a 13-mere (Pilhofer et al. 2011). The evolutionary origin of this bacterial tubulin is still unclear.

#### Health and human interactions

Members of the *Chlamydiae* have been one of the most important pathogens of humankind. Potentially motile chlamydias have recently been described and the role of the flagellar apparatus during infection has been raised (Collingro et al. 2017). In contrast, some members of the *Verrucomicrobia* appear to have beneficial effects (Schneeberger et al. 2015). *Akkermansia muciniphila* is the only cultivated intestinal representative of the *Verrucomicrobia*. Despite the fact that it was isolated from human faecal samples only one decade ago, there has been a growing interest in this organism because of its association with animal and human health. In particular, this genus has been suggested to be a potential biomarker of a healthy gut status (Belzer and de Vos 2012; Derrien et al. 2016). Recently, planctomycetal organisms have been detected by amplification of fragments of their 16S rRNA from wild-gorilla faeces, termite guts and human gut microbiota (Frey et al. 2006; Köhler et al. 2008; Cayrou et al. 2013; Aghnatios and Drancourt 2016). In addition, most planctomycetes have the potential to produce bioactive and antimicrobial molecules (Jeske et al. 2013, 2016; Graça et al. 2016).

#### Earth cycles

Some PVC members are important contributors to major biogeochemical cycles such as the global carbon and nitrogen ones (Fuerst and Sagulenko 2011). Specifically, anammox planctomycetes are major contributors to the global nitrogen cycle (Kuypers et al. 2005; Kartal et al. 2013). On the other hand, some verrucomicrobial species play an important role in the carbon cycle decreasing methane emission.

#### Anammox

Adding to the curiosities of these bacteria, a missing link in a peculiar biochemical reaction was described in a divergent planctomycete. These bacteria perform anaerobic ammonium oxidation using nitrite as the electron acceptor to produce N_2_ gas. Based on biochemical calculations, it had long been predicted that anammox bacteria might contribute approximately 50% of the N_2_ gas released in the marine environment (Brandes et al. 2007). This process has been patented and is now applied at industrial scale in wastewater treatment (Mulder A, 1992, Jetten M., Silvester Maria, Van Loosdrecht Marinus Corneli; Technische Universiteit Delft, Patent WO9807664). This reaction takes place inside the anammoxosome, a membrane defined compartment unique to these bacteria. Possibly one of the first prokaryotic organelle, it was recently isolated and characterized from the anammox bacterium *Kuenenia stuttgartiensis* (Neumann et al. 2014). The anammoxosome membranes with their unique ladderanes lipids are expected to act as a diffusion barrier that confines the toxic intermediates of the reaction and to allow the generation of the proton motive force required for ATP production (Sinninghe Damsté et al. 2002). More work on this “bacterial organelle” is needed.

#### Methylotrophy and methanogenesis

Methanotrophic bacteria are a ubiquitous group of methylotrophic microorganisms that consume methane playing an important role in the control of global warming decreasing methane emission. They are commonly found at the aerobic/anaerobic interfaces of environments such as wetlands, aquatic sediments and landfills, where they consume up to 90% of the methane produced (Segers 1998). Up to the beginning of this century, all known methanotrophic species belonged to the phylum *Proteobacteria*. However, in 2007–2008 the isolation of three thermoacidophilic methanotrophs that represented a distinct lineage within the bacterial phylum *Verrucomicrobia* were described (Dunfield et al. 2007; Pol et al. 2007; Islam et al. 2008). The methanotrophic isolates are so far, the most acidophilic methanotrophs known, with a lower growth limit below pH 1. The isolation of three new species of mesophilic acidophilic verrucomicrobial methanotrophs from a volcanic soil in Italy has been recently reported (van Teeseling et al. 2014). Genomic data of the verrucomicrobial methanotrophs *Methylacidiphilum fumariolicumstrain* SolV and *Methylacidiphilum infernorum* strain V4 also indicated the ability of autotrophic metabolism (Khadem et al. 2011; Sharp et al. 2012).

Genes coding for enzymes involved in methanogenesis or methylotrophy (processes which have common enzymes) have been detected in *Planctomycetes*. No correlation between the presence of these genes and the ability to use or produce C1 compounds, or to detoxify formaldehyde, has been described yet (Chistoserdova et al. 2004; Bauer et al. 2004; Woebken et al. 2007). The functions of these genes are still unknown. The possible contribution of *Planctomycetes* to the origin of methanogenesis or methylotrophy has been highlighted but more research is needed (Chistoserdova et al. 2004).

#### Evolutionary cell biology

Because of the presence of so many different features in related species, PVC bacteria are extremely attractive for the field of evolutionary cell biology. The evolution and divergence from a common ancestor should be deciphered, leading to major advances. How was their diversity generated from the Last PVC Common Ancestor (LPCA)? Are they ancestral bacteria as has been suggested (Brochier and Philippe 2002) or is this an artifact due to long branch attractions? How did the diverse modes of division evolve from a common ancestor dividing by binary fission with PG?

## Future perspectives

One of the hallmarks of the PVC superphylum is that it gathers bacteria with extremely diverse pheno- and genotypes in a group of related ancestry. We are thus in the coveted position in which the characterization of a single feature in a single species could be interpreted in the much broader framework of the whole superphylum encompassing the diversity of variation of this feature, including its absence, duplication, divergence, and replacement of components. We should capitalize on this strength to build ambitious PVC research programs with a global perspective, representing important contributions to the field of evolutionary cell biology.

In conclusion, PVC bacteria are more exciting than ever. The next, fourth, PVC meeting will be held in Nijmegen, the Netherlands in 2019. Are you joining?
